# *Drosophila* caspases as guardians of host-microbe interactions

**DOI:** 10.1038/s41418-022-01038-4

**Published:** 2022-07-09

**Authors:** Christa Kietz, Annika Meinander

**Affiliations:** 1 Faculty of Science and Engineering, Cell Biology, Åbo Akademi University, BioCity, Turku, Finland; 2 InFLAMES Research Flagship Center, Åbo Akademi University, Turku, Finland

**Keywords:** Cell death and immune response, Proteases

## Abstract

An intact cell death machinery is not only crucial for successful embryonic development and tissue homeostasis, but participates also in the defence against pathogens and contributes to a balanced immune response. Centrally involved in the regulation of both cell death and inflammatory immune responses is the evolutionarily conserved family of cysteine proteases named caspases. The *Drosophila melanogaster* genome encodes for seven caspases, several of which display dual functions, participating in apoptotic signalling and beyond. Among the *Drosophila* caspases, the caspase-8 homologue Dredd has a well-characterised role in inflammatory signalling activated by bacterial infections, and functions as a driver of NF-κB-mediated immune responses. Regarding the other *Drosophila* caspases, studies focusing on tissue-specific immune signalling and host-microbe interactions have recently revealed immunoregulatory functions of the initiator caspase Dronc and the effector caspase Drice. The aim of this review is to give an overview of the signalling cascades involved in the *Drosophila* humoral innate immune response against pathogens and of their caspase-mediated regulation. Furthermore, the apoptotic role of caspases during antibacterial and antiviral immune activation will be discussed.

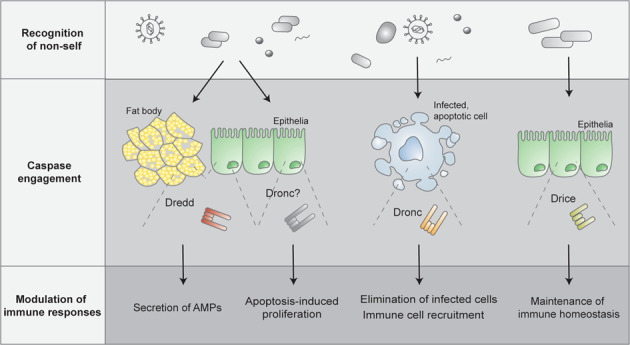

## Introduction

**Fig. 1:**
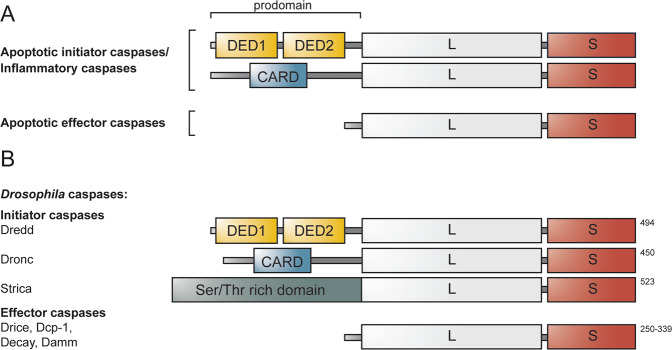
General domain architecture of caspases, and classification of the caspases identified in *Drosophila*. **A** Based on their described function, caspases are divided into inflammatory and apoptotic caspases. The apoptotic caspases are further subdivided into initiator and effector caspases. Caspases contain a small (S) and a large (L) subunit that together form the protease domain. In addition, inflammatory and apoptotic initiator caspases contain an N-terminal long prodomain harbouring DED or CARD domains, whereas apoptotic effector have short prodomains lacking specific protein domains. **B** *Drosophila melanogaster* carries three initiator caspases: Dredd, Dronc and Strica. In contrast to Dredd and Dronc, the atypical initiator caspase Strica does not contain DED or CARD domains, but harbours instead a Ser/Thr rich prodomain. In addition to the initiator caspases, *Drosophila* carries four effector caspases, Drice, Dcp-1, Decay and Damm. The length of the caspases in amino acids are indicated to the right of the proteins.

Cell death and inflammation are cellular processes crucial for maintaining tissue homeostasis [1]. Centrally involved in both processes is the evolutionarily conserved family of cysteine proteases, named caspases (cysteine-aspartic proteases). By driving apoptosis, a homeostatic form of cell death, caspases control the non-lytic elimination of cells during development, and the clearance of cells that are damaged, old or no longer necessary in the adult organism [2]. Besides regulating apoptosis, caspases also drive inflammatory signalling in response to pathogenic infection by triggering the release of inflammatory cytokines and by inducing pyroptosis, a proinflammatory form of cell death [2, 3]. Based on these described functions, caspases can be broadly divided into inflammatory and apoptotic caspases. The apoptotic caspases are further subdivided into initiator and effector caspases, depending on their position in the apoptotic signalling cascade [3]. Structurally, caspases consist of an amino-terminal prodomain of variable size followed by one large (p20) and one small (p10) subunit that together form the catalytically active protease domain (Fig. 1A). Inflammatory and apoptotic initiator caspases contain specific recruitment domains, i.e., Death effector domains (DEDs) or Caspase recruitment domains (CARDs) in the N-terminal prodomain (Fig. 1A). These domains facilitate recruitment of caspase monomers to oligomeric activation platforms, e.g., the pattern-recognition receptor (PRR) induced inflammasomes, the mammalian apoptosome, the Death inducing signalling complex (DISC), and the p53-induced protein with a death domain (PIDD)-osome, in which the initiator caspases are activated [4–6]. The apoptotic effector proteins have short prodomains that lack specific interaction domains (Fig. 1A). These caspases exist as dimers and gain activity through proteolytic processing of a linker region separating the large and small subunit, mediated by an upstream caspase [6, 7].

Given their crucial role as executors of cell death, both the activation process and the enzymatic activity of caspases need to be carefully regulated. In addition to synthesising caspases as inactive zymogens, or procaspases, requiring dimerization and proteolytic processing to gain activity, the cell employs several strategies, such as decoy proteins, posttranslational modifications and caspase inhibitors to regulate caspase activity [7, 8]. In metazoans, the main protein group of caspase regulators is the Inhibitor of apoptosis (IAP) protein family, whose members harbour one to three characteristic Baculoviral IAP repeat (BIR) domains [9]. Some IAPs are furthermore, important transduction intermediates in cellular signalling cascades, specifically during innate immune responses and Nuclear factor κB (NF-κB) activation [10, 11]. Due to the role of caspases and IAPs at the frontline of immunity and cell death, these proteins and their interaction have served as interesting targets when studying inflammatory signalling and immunohomeostasis.

*Drosophila melanogaster* is one of the most commonly used model organisms in biological research. Its short life cycle and high breeding rate, low maintenance fees, and a simpler, less redundant genome compared to mammalian models, have made the fruit fly an invaluable research model [12]. Moreover, the versatile genetic tool-box of *Drosophil*a with collections of mutants and genetically modified flies, engineered to enable manipulation of gene activity both temporally and spatially, enables sophisticated genetic analyses to be carried out on tissue or whole-organism level in the fly. Research done in *Drosophila* has contributed to major advancements in the fields of genetics, development, behaviour and immunity [13, 14] and, the fruit fly is recognised as a powerful disease model for diabetes, cancer, and neuropathologies, and for the distinct diseases of heart, lung and intestine [15–20]. When it comes to the cellular regulation of innate immunity, research performed in *Drosophila* has contributed greatly in understanding receptor activation, signal transduction and transcriptional activation during host defence [21]. Similarly, the molecular role of caspases in innate immunity, inflammatory signalling and immunohomeostasis has been elucidated in the fly [22–27]. In addition to the molecular regulation of inflammation, *Drosophila* has emerged as a versatile model in which to study complex physiological aspects of immune defence, such as the spread or restriction of infection [28], the priming and memory of the immune system [29] and, finally, the local immune system and microenvironment of barrier epithelia [30]. This review aims to give an overview of the structure and function of the *Drosophila* caspases and of the humoral part of the fly’s immune system. It will describe the current knowledge on caspase-mediated regulation of inflammatory signalling induced upon infection and during host-microbe interactions. In addition, the role of cell death activation in response to viral and bacterial infection will be discussed.

## *Drosophila* caspases and their function

Caspases have been identified in all metazoans, ranging from *Caenorhabditis elegans* and *Drosophila*, to mouse and human [31]. *Drosophila* carries seven caspases: three initiator caspases, namely Death related ced-3/Nedd2-like caspase (Dredd), Death regulator Nedd2-like caspase (Dronc) and Ser/Thr-rich caspase (Strica) [32–34], and four effector caspases, called *Drosophila* caspase interleukin 1β-converting enzyme (Drice), Death-associated molecule related to Mch2 (Damm), Death executioner caspase related to apopain/yama (Decay) and *Drosophila* caspase-1 (Dcp-1) [34–37] (Fig. 1B). All *Drosophila* caspases have been connected to apoptotic signalling [32, 35–40], however, some seem to have their main function, or additional roles, beyond cell death [22, 24, 41–43].

*Drosophila* Dronc is homologous to human caspase-9 and is the main apoptosis-initiating caspase in the fly [44]. In resting cells, Dronc is inhibited by the antiapoptotic protein *Drosophila* iap1 (Diap1) [45]. During apoptosis, the proapoptotic proteins Reaper, Grim and Hid bind to Diap1, antagonising the Diap1-Dronc interaction, hence freeing Dronc [46, 47]. Freed Dronc is recruited to Death-associated Apaf1-related killer (Dark) via CARD-CARD interactions and is subsequently activated [48, 49]. Activated Dronc cleaves effector caspases Drice and Dcp-1, which in turn cleave downstream substrates, thereby executing apoptosis [38, 45]. In addition to inhibiting Dronc, Diap1 has been shown to inhibit Drice and Dcp-1 [47, 50]. These effector caspases are homologous to mammalian caspase-3 and seem to have partially overlapping functions during apoptosis. Dcp-1 mutants display milder defects in apoptotic signalling compared to Drice mutants, however, the phenotype of double Drice/Dcp-1 mutants is stronger than that of either one alone [43, 51, 52]. A second *Drosophila* IAP protein, *Drosophila* iap2 (Diap2), mainly known for its potent role as an inducer of inflammatory signalling upon infection by Gram-negative bacteria [53–56], has also been shown to inhibit Drice, thereby lowering the apoptotic threshold of the cell [57]. We, furthermore, recently demonstrated that Drice, through its interaction with Diap2, has a role beyond apoptotic signalling as a regulator of inflammatory signalling [24].

While the caspase-8 homologue Dredd has been implicated to function in apoptotic signalling [32, 58], its major function has been established to be a regulator of the inflammatory response triggered by Gram-negative bacteria [22]. Dredd contains two DED domains in its prodomain, needed for caspase recruitment to the bacteria-induced receptor complex and for interaction with Diap2 [23, 59]. Besides being homologous to caspase-8, Dredd is also structurally similar to cellular FLICE-like inhibitory protein (c-FLIP), a member of the mammalian DISC-complex that regulates both apoptotic caspase-8 activity and inflammatory NF-κB signalling [60]. Similarly as caspase-8 and Dredd, c-FLIP contains two DED domains that mediate recruitment to DISC, and is suggested to facilitate interaction with the downstream NF-κB pathway member NF-κB essential modulator (NEMO) [60, 61]. Hence, it seems that the functions performed by Dredd have evolved to be executed separately by caspase-8 and c-FLIP in mammals. The caspases Decay, Strica and Damm have not received as much attention as the other *Drosophila* caspases, however, Decay was recently found to regulate wing size, independently of Dronc-induced apoptosis [62]. Strica is known to contain a unique serine and threonine rich prodomain, however, the functions of Strica or Damm, remain largely unknown.

## The *Drosophila* immune response

**Fig. 2:**
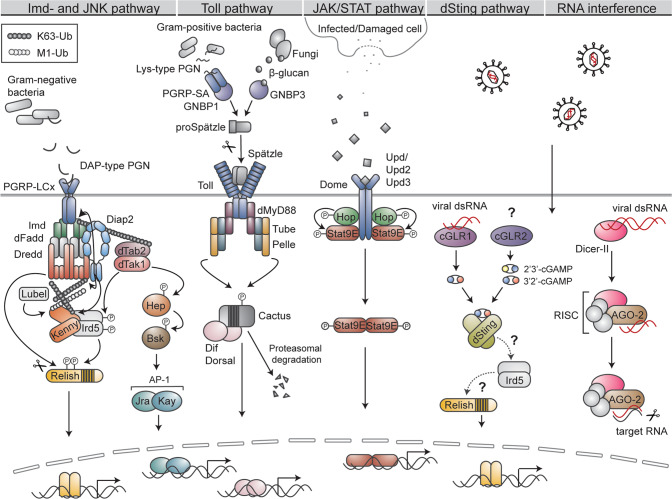
Signalling pathways regulating *Drosophila* innate immunity. The Imd pathway is initiated by DAP-type PGN derived from Gram-negative bacteria binding to the PGRP-LC receptor. The adaptor proteins Imd and dFadd, the caspase Dredd and the E3 ligase Diap2 are recruited to the receptor, whereafter Diap2 ubiquitinates Imd, Dredd and Kenny with K63-linked ubiquitin chains. The chains on Imd are thought to recruit the dTak1/dTab2 complex, whereas the chains on Dredd are needed for caspase activity and Relish cleavage. In addition to K63-linked chains, Kenny is also modified by M1-linked ubiquitin chains synthesised by Lubel. The dTak1/dTab2 complex is upstream of the IKK complex, consisting of Kenny and Ird5, which activates Relish by Ird5-mediated phosphorylation. The pathway culminates in translocation of Relish to the nucleus and target gene expression. In addition to driving Imd signalling, dTak1 functions as one of the apical-most kinases in the JNK pathway. Activated dTak1 phosphorylates Hep, that in turn phosphorylates Bsk. Bsk activates the transcription factor complex AP-1, consisting of Kay and Jra, which drives target gene expression after its nuclear translocation. The Toll pathway is induced when Lys-type PGN, originating from the cell wall of Gram-positive bacteria, is recognised by the PGRP-SA/GNBP1 receptor complex or when β-glucan, derived from fungi, is sensed by GNBP3. The activated receptors induce a serine-cascade culminating in the maturation of the ligand Spätzle from proSpätzle, whereafter Spätzle is recognized by the Toll receptor. Receptor activation leads to the recruitment of dMyD88, Tube and Pelle and subsequent Pelle-mediated phosphorylation of the IκB protein Cactus, targeting the protein for proteasomal degradation. Freed transcription factors Dif and Dorsal translocate to the nucleus and drive target gene expression. JAK/STAT signalling is activated by the cytokines Upd, Upd2 and Upd3, secreted by infected or damaged neighbouring cells. The Upds bind the receptor Dome, leading to phosphorylation of receptor-associated Hop. Activated Hop phosphorylates the transcription factor Stat9E, hence inducing its dimerisation and nuclear translocation. The dSting pathway is activated by viral dsRNA, sensed by the receptor cGLR1. The activated receptor produces 3’2’-cGAMP, a secondary messenger activating dSting. Signalling downstream of dSting remains largely elusive, however Ird5 and Relish seem to be needed for dSting target gene expression. In addition to cGLR1, cGLR2 is known to produce both 2’3’-cGAMP and 3’2’-cGAMP, however, its upstream trigger remains unknown. Viral dsRNA is recognised and cut into smaller fragments by Dicer-II. The fragments are loaded into AGO-2-containing RNA-induced silencing complex (RISC) wherein one of the viral RNA strands is degraded. The activated RISC complex then recognises and degrades RNA containing complementary sequences to the original viral RNA.

In its defence against pathogens, *Drosophila* mainly relies on an innate immune response, aided, similarly as in mammals, by physical barriers, such as the epithelial lining beneath the cuticle in the digestive tract and trachea [21]. The *Drosophila* innate immune system can be roughly divided into a humoral and a cellular response. The cell-mediated immune response of *Drosophila* is carried out by freely circulating, or tissue-associated specialised blood cells, called haemocytes [63]. The haemocytes participate in the immune response by mediating phagocytosis, encapsulation of invading pathogens, wound closure, and by secretion of clotting factors and cytokines [63]. These cells also function as activators of the humoral part of the immune system, and the crosstalk between the cellular and humoral response is abundant [63, 64]. The humoral response involves the production of antimicrobial peptides (AMPs) and antipathogenic factors through the Toll, Imd, c-Jun N-terminal kinase (JNK) and Janus kinase/signal transducer and activator of transcription (JAK/STAT) signalling pathways (Fig. 2) [21]. The aforementioned pathways seem to also be involved in the antiviral defence. However, activity of this fraction of the immune response is suggested to mainly be mediated via the RNA interference (RNAi) pathways, restricting viral replication via targeted degradation of viral double stranded RNA (dsRNA) [65, 66]. In addition to RNAi, the *Drosophila* stimulator of interferon genes (dSting) pathway, inducing expression of antiviral factors upon sensing of viral dsRNA by the cyclic GMP-AMP (cGAMP) synthase (cGAS)-like receptor 1 (cGLR1), aids in the immune response against viruses (Fig. 2) [67]. The presence of an adaptive immune response in insects remains relatively unexplored. However, described cases of immune priming, resulting in a stronger specific immune response towards secondary infection [68] and RNAi-based immunological memory [69], point towards the presence of a specific, adaptive immune response also in *Drosophila*.

## Caspase-mediated regulation of the Imd pathway

**Fig. 3:**
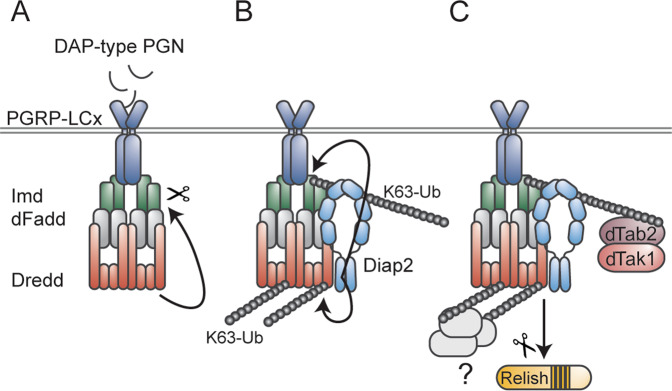
Dredd drives Imd signalling and Relish activation. **A** Upon activation by DAP-type peptidoglycan, Imd and dFadd are recruited to the PGRP-LC receptor. Dredd binds to dFadd, is activated, and cleaves Imd. **B** Cleavage of Imd enables recruitment of Diap2 that also interacts directly with Dredd. Diap2 catalyses the formation of K63-linked ubiquitin chains on Dredd and Imd. **C** The K63-linked chains on Imd are thought to recruit the dTak1/dTab2 complex, and the chains on Dredd might function as a scaffold for additional proteins or protein complexes needed for downstream signalling. Ubiquitination of Dredd is, in addition, needed for proteolytic processing and nuclear translocation of Relish.

A hallmark of the *Drosophila* innate immune response is the Imd and Toll pathway-mediated activation of NF-κB transcription factors that drive the production and secretion of AMPs (Fig. 2) [21]. The Imd pathway is activated by diaminopimelic acid (DAP)-type peptidoglycan (PGN), present in the cell wall of Gram-negative bacteria, recognised by the transmembrane PRR Peptidoglycan recognition protein (PGRP)-LC or the intracellular receptor PGRP-LE [70–73]. The receptors are thought to dimerise or oligomerise upon ligand binding, whereafter the adaptor protein Imd is recruited to the complex [74–76]. Imd recruits the adaptor protein *Drosophila* Fas-associated death domain protein (dFadd), which in turn binds to the caspase-8 homologue Dredd [59, 77], a central component of the Imd pathway, and a driver of NF-κB-mediated immune responses (Fig. 3). The importance of Dredd in the *Drosophila* immune response was first demonstrated in 2000, when Lemaitre and colleagues identified five Ethyl methanesulfonate-induced mutations in Dredd, all severely impairing bacteria-induced *Diptericin* expression in the fly [22]. Further characterisation of one of the mutants, Dredd^B118^, containing a premature stop-codon in the Dredd prodomain, revealed the caspase to be crucial specifically during the immune response induced by Gram-negative bacteria [22]. The link between Dredd and activation of the NF-κB transcription factor Relish was further elucidated by the laboratory of Dan Hultmark, showing that activation of Relish proceeds through a signal dependent endoproteolytic step leading to the nuclear translocalisation of the Rel-68 truncated form, and that this Relish-cleavage was absent in Dredd mutants post infection [27]. Dredd was later confirmed to, indeed, be the caspase responsible of cleaving Relish [78, 79], but also to process the apical-most adaptor protein of the Imd pathway, i.e., Imd itself [26, 79], and to, thereby, enable recruitment of Diap2 to the receptor complex (Fig. 3A, B) [26]. By studying the Diap2-Dredd interaction and characterising one of the Dredd mutants originally identified by Leulier et al., Dredd^D44^, harbouring a glycine-to-arginine point mutation at position 120 [22], we were able to show that signal-dependent lysine 63 (K63)-linked ubiquitination of Dredd is required for Relish target gene activation (Fig. 3C), and for fly survival in response to Gram-negative bacterial infection [23]. The function of the ubiquitin chains on Dredd remains to be elucidated. However, it is possible that these chains serve as scaffolds for the recruitment of other protein complexes needed for downstream signalling, such as the IκB kinase (IKK) complex, consisting of the regulatory subunit Kenny, homologous to mammalian NEMO or IKKγ, and of the catalytic subunit called Immune response deficient 5 (Ird5), homologous to the mammalian IKKβ [80, 81]. Furthermore, as synthesis of methionine 1 (M1)-linked ubiquitin chains is required for local NF-κB-mediated immune responses in the fly [82], ubiquitin chain types beyond the previously described K63-linked chains might regulate yet unidentified tissue specific functions on Dredd. In addition to Dredd-mediated activation of Relish and recruitment of the aforementioned IKK complex, the *Drosophila* TGF-β activated kinase 1 (dTak1)/*Drosophila* Tak1 binding protein (dTab2) complex is needed for intact Imd signalling [74, 78]. The role of dTak1 may be to induce activation of Ird5 by phosphorylation, similarly as Ird5 is known to activate Relish by phosphorylation. However, while Relish phosphorylation drives transcription and recruitment of RNA polymerase II, it is not needed for Relish cleavage or nuclear translocation [78].

**Fig. 4:**
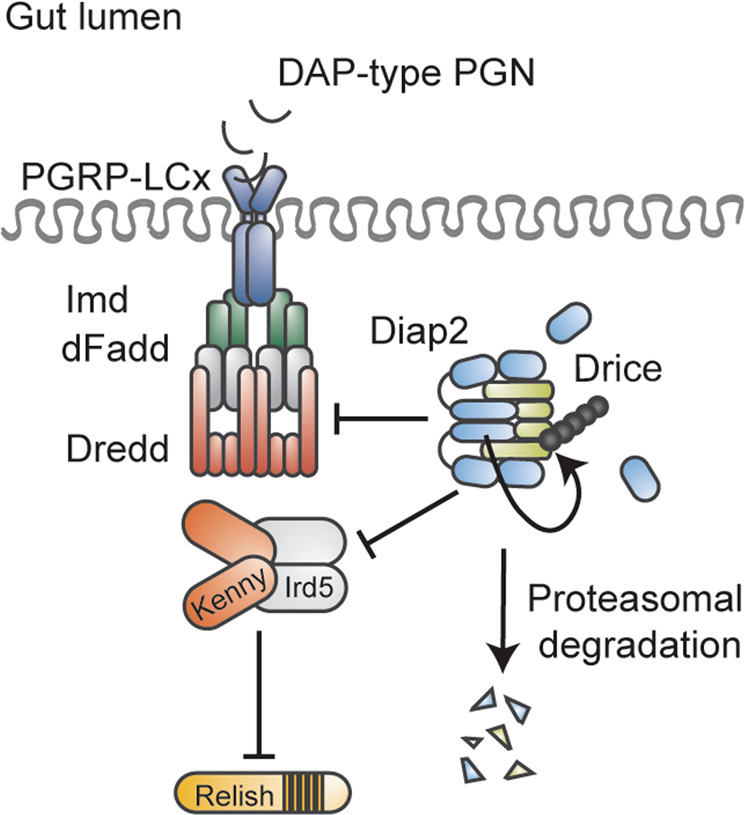
Drice restrains Imd signalling induced by commensal bacteria. During basal conditions, DAP-type PGN originating from commensal bacteria in the gut lumen activates the PGRP-LC receptor, leading to the recruitment of Imd, dFadd and Dredd. The receptor complex aims at recruiting Diap2 to drive downstream signalling. Drice halts Imd signalling by forming an inhibitory complex with Diap2, targeted for proteasomal degradation, interfering, hence, with the ability of Diap2 to interact with pathway members and activate downstream signalling.

In contrast to the fat body-mediated immune response towards bacterial infections, regulated by both the Imd and Toll pathway, Imd signalling is believed to be the sole driver of NF-κB activity during local epithelial immune responses of, for instance, the gut and trachea [83, 84]. Intestinal Imd signalling needs to be carefully regulated in order to ensure efficient elimination of pathogens, while allowing for beneficial host-microbe interactions to be established. We have recently described a role of the caspase Drice as a negative regulator of intestinal Imd signalling induced by commensal bacteria [24]. By forming a covalent complex with Diap2, the details of which have been previously described [57], Drice triggers the tissue-specific proteasomal degradation of both proteins [24]. As a consequence, Diap2 is unable to interact with members of the Imd pathway and downstream signalling is halted (Fig. 4). As Diap2 has been shown to ubiquitinate itself [23] and Drice [57], we speculate that formation of a Diap2-Drice complex induces Diap2-mediated K48-linked ubiquitination of both proteins, targeting them for proteasomal degradation (Fig. 4). Drice-mediated regulation of Diap2 indicates that caspases, known to be regulated by IAP-proteins during cell death [8], are indeed themselves able to modify the activity of inflammatory IAP-proteins.

## The *Drosophila* Toll signalling pathway

Toll signalling is initiated by extracellular PRRs that recognise conserved structures of the cell wall of fungi or Gram-positive bacteria. Fungal β-glucan is identified by the Gram-negative binding protein 3 (GNBP3) [85], whereas Lys-type PGN from Gram-positive bacteria is identified by a complex consisting of PGRP-SA and GNBP1 (Fig. 2) [86]. These PRRs induce a serine-cascade leading to the proteolysis-dependent activation of the extracellular cytokine Spätzle that, by functioning as the Toll receptor ligand, activates downstream signalling [87, 88]. Upon receptor activation, the adaptor protein Myeloid differentiation primary response (MyD88), the adaptor protein Tube and the kinase Pelle are recruited to form a MyD88-Tube-Pelle trimeric complex [89, 90]. Pelle drives downstream signalling by phosphorylating the inhibitory IκB protein Cactus, thereby targeting Cactus for proteasomal degradation [91]. The NF-κB transcription factors Dorsal-related immunity factor (Dif) and Dorsal, sequestered in the cytoplasm by Cactus in resting cells, are released, and enter the nucleus in order to activate anti-inflammatory target genes [91–93]. Among the *Drosophila* caspases, Dronc and Dredd have been connected to the Toll-pathway. Apoptosis-deficient Dronc mutants display chronic activation of Toll signalling in the absence of infection, and the caspase has been suggested to be involved in the regulation of the Toll-mediated inflammatory response towards danger-associated molecular patterns (DAMPs) [94]. In addition to binding Tube and Pelle, dMyD88 has been shown to interact with dFadd when overexpressed in *Drosophila* S2 cells [95]. As dFadd is known to recruit Dredd during Imd signalling, this result may indicate that Dredd is able to regulate immune responses induced by Gram-positive bacteria in certain tissues, or, conversely that members of the Toll pathway are recruited downstream of dFadd during activation of the PGRP-LC/LE complex. Although a functional role of Dredd in Toll signalling is yet to be demonstrated, the caspase serves as an interesting candidate when studying mediators of crosstalk between the two NF-κB pathways.

## Dredd is needed for bacteria-induced JNK signalling

Besides driving Imd signalling, dTak1 functions also as one of the apical-most kinases in the conserved JNK pathway (Fig. 2) [96]. JNK is involved in a variety of biological processes in *Drosophila*, including development, metabolism and apoptosis, and in stress and immune responses [97]. Upon activation of dTak1, the kinase phosphorylates the JNK kinase Hemipterous (Hep), which in turn phosphorylates the single *Drosophila* JNK protein Basket (Bsk) [98, 99]. The JNK pathway culminates, depending on the cellular setting, in the activation of the transcription factors Forkhead Box O (FOXO), or Activator protein-1 (AP-1), a heterodimer consisting of Jun-related antigen (Jra), homologous to mammalian c-Jun, and Kayak (Kay), homologous to mammalian Fos, and to subsequent target gene expression [97]. Co-regulation of JNK and NF-κB signalling is required for a balanced immune response and JNK is needed for proper release of AMPs and is, furthermore, required for Imd-induced epithelial shedding [100, 101]. In addition, the expression patterns of JNK are regulated by Relish that induces the proteasomal degradation of dTak1 upon activation by a Gram-negative bacterial infection, hence terminating JNK signalling [102]. Similarly as for the Imd pathway, Dredd has been shown to be needed for bacteria-induced JNK signalling. RNAi-mediated downregulation of the caspase in *Drosophila* S2 cells impairs phosphorylation of JNK and subsequent JNK target gene expression upon PGN treatment [103, 104]. Concordantly, Dredd^B118^ mutants are unable to induce JNK phosphorylation or target gene expression in response to septic infection with *E. coli* [104].

## Caspases regulating the *Drosophila* antiviral immune response

In its defence against viral infections, *Drosophila* relies on the antiviral RNAi system and on inducible responses mediated via the dSting, JAK-STAT, Toll and Imd pathways [105]. The host RNAi pathway is triggered upon sensing of viral dsRNA by the RNase III enzyme Dicer-II that processes the dsRNA into small interfering RNAs (siRNA). The siRNAs are loaded into the Argonaute-2 (AGO2) protein, part of the RNA-induced silencing complex (RISC) and guide AGO2 to target RNAs to induce their degradation, hence, restricting viral replication (Fig. 2) [65, 66]. Another sensor of viral dsRNA is cGLR1, which drives dSting signalling in virus infected cells. The recognition of viral RNA, triggers cGLR1-mediated synthesis of the secondary messenger 3’2’-cGAMP that by interacting with dSting, drives the dSting-dependent antiviral immune response (Fig. 2) [106, 107]. Interestingly, a second dSting-activating cGLR, cGLR2, producing both 2’3’-cGAMP and 3’2’-cGAMP, was identified simultaneously as cGLR1, however, its upstream ligand remains unidentified [107].

The JAK/STAT pathway is, similarly as in the mammalian host defence, a key regulator of the *Drosophila* immune response against virus infections [108, 109]. JAK/STAT signalling contributes, however, also to the immune defence against bacterial infections and controls cellular immunity by regulating haemocyte proliferation and differentiation [110–112]. The pathway is driven by three cytokine-like proteins: Unpaired (Upd), Upd2 and Upd3, expressed to various extents during development, tissue damage, viral infections and bacterial challenge [110, 113, 114]. The Upds bind to the receptor Domeless (Dome), inducing its dimerisation [115] and subsequent activation of the receptor-associated JAK homologue Hopscotch (Hop) [116]. Hop phosphorylates Stat9E, a homologue of the mammalian transcription factor STAT [117] that dimerises, translocates to the nucleus and drives target gene expression (Fig. 2). In response to viral infections, JAK/STAT target genes include antiviral effectors such as *TurandotM (TotM*) and *virus-induced RNA-1* (*vir-1*) [113, 118, 119]. Interestingly, activation of JAK/STAT signalling does not seem to be a general defence mechanism during viral infection, but is induced only in response to specific viruses [112, 113, 119].

Regarding the function of Toll and Imd signalling during antiviral immune responses, mutants of signalling mediators of both pathways have been shown to display increased susceptibility to viral infections. However, the details of receptor activation, virus specificity and number of pathway members involved remains to be elucidated [120–123]. Further strengthening the role of NF-κB signalling as an important factor during antiviral defence, is the identification of viral suppressors of Toll and Imd signalling in the in the genomes of Kallithea viruses and Invertebrate Iridescent Virus-6 (IIV-6), respectively [124, 125]. Similarly, homologues of a cytokine named Diedel (Die), up-regulated in *Drosophila* upon certain viral infections and proposed to protect the fly from detrimental consequences by preventing excessive activation of Imd signalling, are encoded by insect DNA viruses, hence indicating an evolved need of viruses to suppress *Drosophila* NF-κB signalling [126]. When it comes to the caspase-mediated regulation of the RNAi system, the JAK-STAT pathway, and the Imd and Toll pathways in response to viral infection, little is known. However, as Relish mutants display an impaired antiviral immune response [122–124], it is tempting to speculate that Dredd, given its role as an activator of Relish, also plays a role in Imd signalling during antiviral defences. Interestingly, in a study conducted by Imler and colleagues, ectopic expression of dSting was shown to prevent viral replication in a Ird5- and Relish-dependent manner. In this study RNAi-mediated silencing of Dredd in S2 cells led to a small increase in *Drosophila* C virus replication [127]. However, as no definite conclusions of Dredd regulating dSting can be drawn from this study, further in vivo characterization of Dredd mutants upon viral infection are needed to elucidate a potential role of Dredd in the dSting-Relish axis.

## Apoptosis modulating *Drosophila* innate immune defence

In addition to being an efficient eliminator of damaged or unnecessary cells, apoptosis contributes to a well-functioning immune defence and is often induced upon pathogenic infections [128]. Apoptotic cells facilitate their own removal by recruiting phagocytes, leading to the elimination of infected host cells in a controlled manner, and prevention of a possible spread of the infectious agent [129, 130]. Phagocytosis of infected cells contributes to the successful elimination of viruses in both *Drosophila* and mammals [123, 131, 132]. Upon virus infection in fly cells, apoptosis is induced as a consequence of a decrease in Diap1 levels that leads to the increase of active Dronc and Drice [131, 133]. As phagocytic clearance of virus-infected *Drosophila* S2 cells has been shown to depend on caspase activation [132], apoptosis seems to be a trigger of phagocytosis during the *Drosophila* immune response.

In order to maintain tissue homeostasis upon cell death activation, apoptotic cells induce Dronc-dependent compensatory proliferation of neighbouring healthy cells [51, 134, 135]. Studies performed in the epithelial cells of the imaginal discs, indicate that Dronc would drive apoptosis-induced proliferation [41] and neoplastic activity [41, 136] by stimulating the production of reactive oxygen species (ROS), hence, attracting hemocytes that by cytokine secretion activate epithelial JNK signalling and drive proliferation. An organ naturally subjected to continuous cell turnover is the *Drosophila* midgut [137, 138]. Enterocyte cell death and caspase activity have been shown to influence intestinal stem cell proliferation and are needed for maintaining homeostatic renewal of cells [25, 139]. Of the *Drosophila* caspases, Dronc is known to regulate enterocyte turnover and seem to, depending on the cellular setting, be either limiting or driving intestinal stem cell activity [42, 140]. The epithelial turnover itself is affected by the metabolic state of the fly, but also by external factors such as pathogenic bacteria [111, 141–143]. Local bacterial insults are associated with increased caspase activity and cell death, as well as with higher amounts of ROS that, although contributing to the elimination of bacteria, also harms the epithelial cells [111, 144]. To overcome the damage inflicted by bacteria, compromised cells produce Upds that activate JAK/STAT signalling, driving compensatory stem cell-mediated proliferation [111, 142, 144]. The function of specific caspases in intestinal epithelial regeneration upon bacterial infection remains unexplored. However, given the role of Dronc as a regulator of epithelial proliferation, it is likely that the caspase also contributes to epithelial regeneration in the *Drosophila* intestine during local bacterial insults. Indeed, loss-of-function Dark mutants, unable to activate Dronc, were shown to display increased sensitivity to wounding, due to inability of driving caspase activation and tissue regeneration in the midgut [25].

In addition to Dronc, the effector caspase Drice seems to be activated in the intestinal enterocytes upon bacterial infection. Indeed, effector caspase activation, presumably by Drice, has been used as a marker of apoptosis in the *Drosophila* midgut post infection [111, 143]. As Drice levels are increased upon bacterial infections, the Drice-Diap2 complex described earlier (Fig. 4), might, besides regulating Diap2 and NF-κB-mediated immune signalling [24], also play a role in maintaining homeostatic cell turnover by restraining excessive Drice activity, and apoptosis-mediated cell proliferation during steady-state conditions.

## Conclusion and future perspective

Since their initial discovery in *C. elegans*, caspases have been the subject of intense research, both in the field of programmed cell death and as regulators of inflammatory signalling [3, 145]. Although traditionally separated into inflammatory and apoptotic caspases, overlapping functions of the mammalian caspases from both categories have become evident. Inflammatory caspase-1 has, for instance, been shown to engage apoptotic effector caspase-7 in *S. typhimurium*-infected macrophages [146], and mammalian caspase-8, best known for its initiating role in the extrinsic apoptotic pathway, is able to regulate the Nod-like receptor family pyrin domain containing 3 (NLRP3) inflammasome, to cleave proIL-1β and to drive NF-κB signalling during specific cellular conditions [147]. Given their central role in tissue health and homeostasis, caspases serve as interesting targets when tuning inflammatory signalling. In addition, mutations affecting caspases and their signalling pathways are connected to severe autoimmune and autoinflammatory diseases [148, 149]. In order to treat these conditions and to find potential therapeutic targets, the cellular mechanisms of caspase regulation and, importantly, the dual function of caspases in cell death and immune defence needs to be elucidated.

Studies done in *Drosophila* during the last two decades have contributed greatly to our knowledge regarding caspase-mediated regulation of immune signalling and epithelial immunohomeostasis. Although the function of Dredd in the immune response towards Gram-negative bacteria has been elucidated in considerable detail, several questions regarding Dredd as a regulator of innate immunity remain to be addressed. Among these are the function of Dredd during virus immune responses, the role of Dredd-ubiquitination in inflammatory signalling, and whether ubiquitin-patterns vary during septic and local immune responses, and, finally, the involvement of Dredd in the response towards Gram-positive bacteria in the intestinal epithelia. Indeed, the recently reported tissue-specific function of Drice in intestinal immunity [24], underscores the difference in inflammatory regulation during acute, septic immune responses and local immune responses induced in tissues that are exposed to both resident and pathogenic bacteria during normal life. To expand our understanding of caspase-mediated regulation of the immune defence in the intestinal epithelia, further studies regarding the upstream drivers of Drice activity, and the functions of Dronc during steady-state conditions and in response to bacterial insults, are needed. Finally, as the potential immune-regulatory functions of Strica, Dcp-1, Damm and Decay remain elusive, further characterisation of these caspases will likely provide the field with valuable knowledge regarding caspase-mediated regulation of innate immunity.

### Reporting summary

Further information on research design is available in the Nature Research Reporting Summary linked to this article.

## Supplementary information

Reporting Summary
